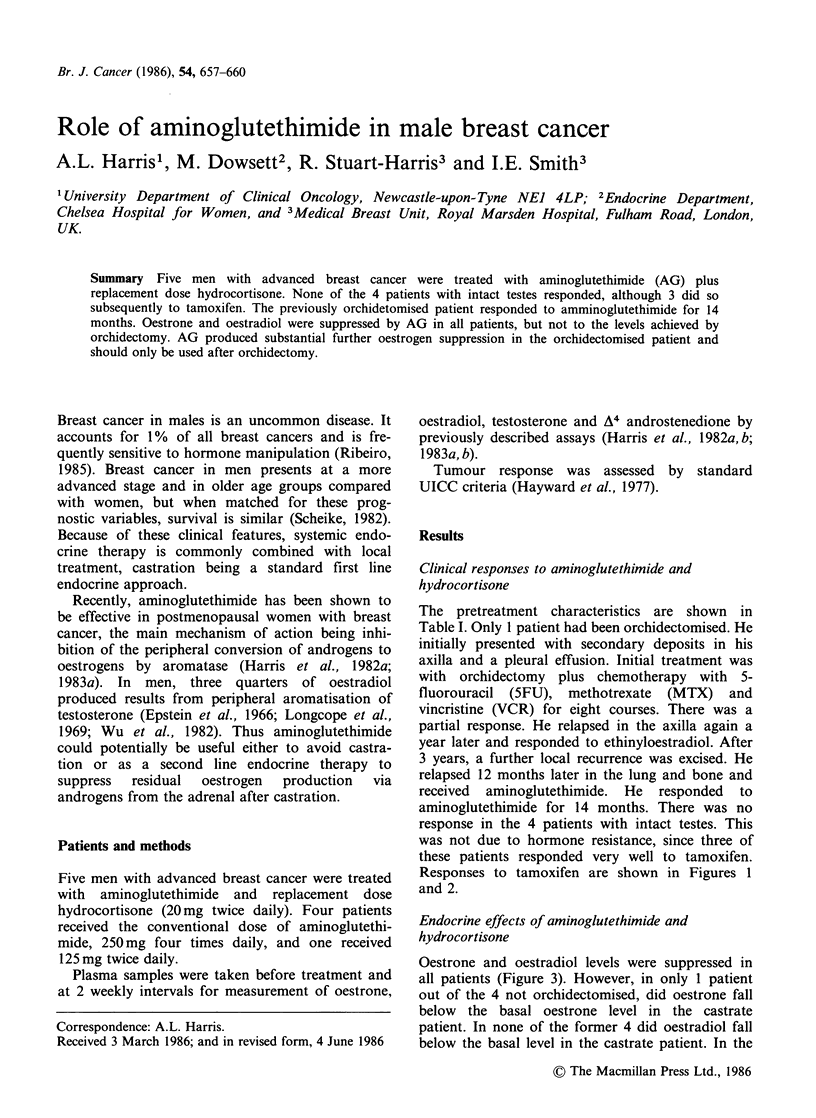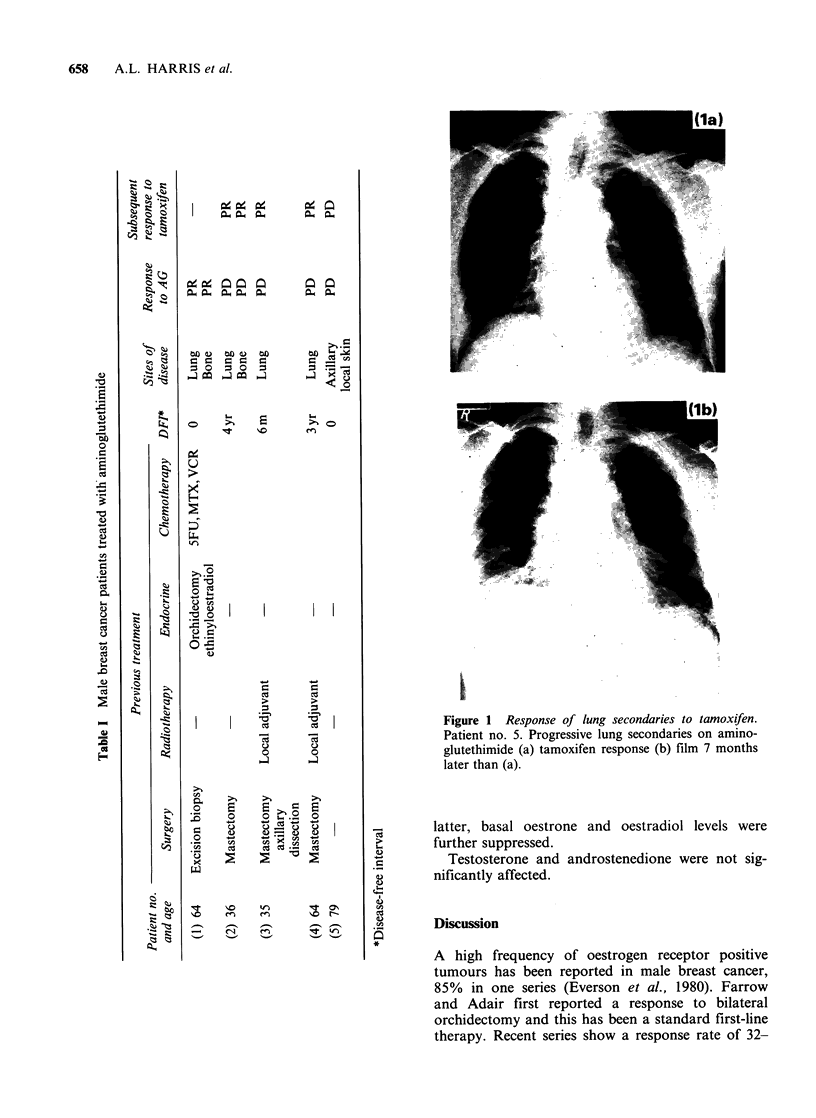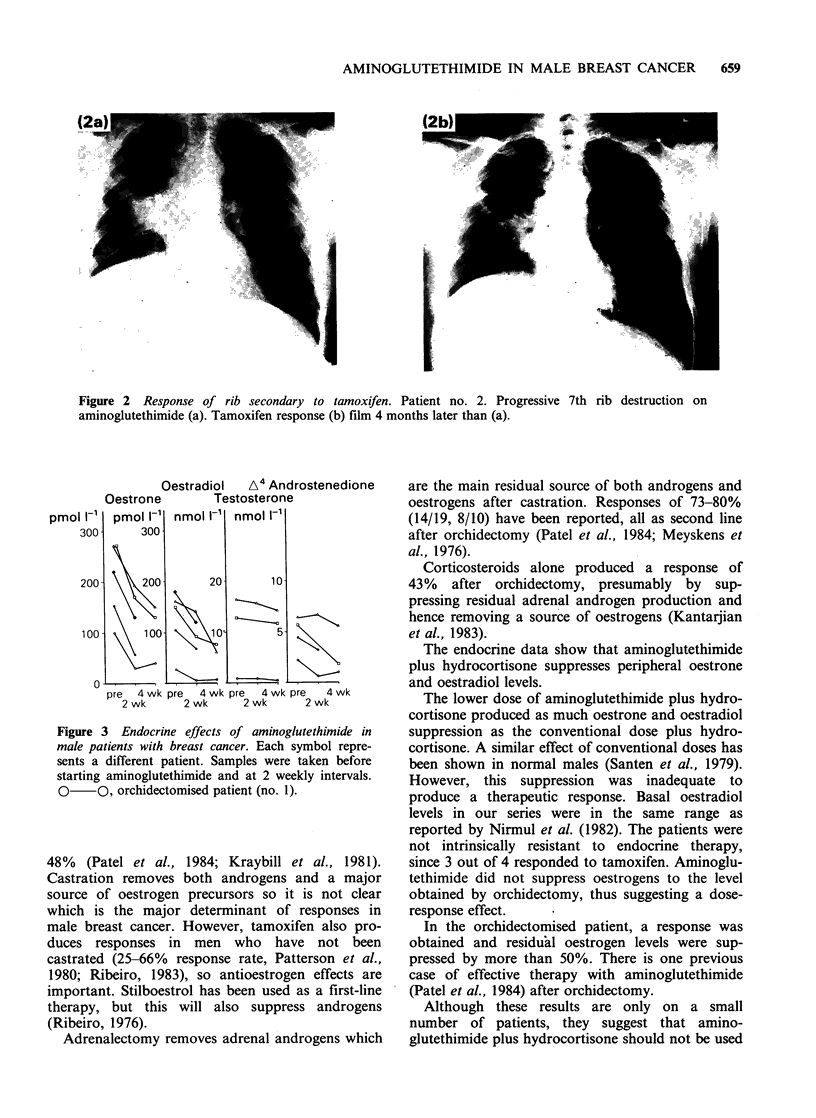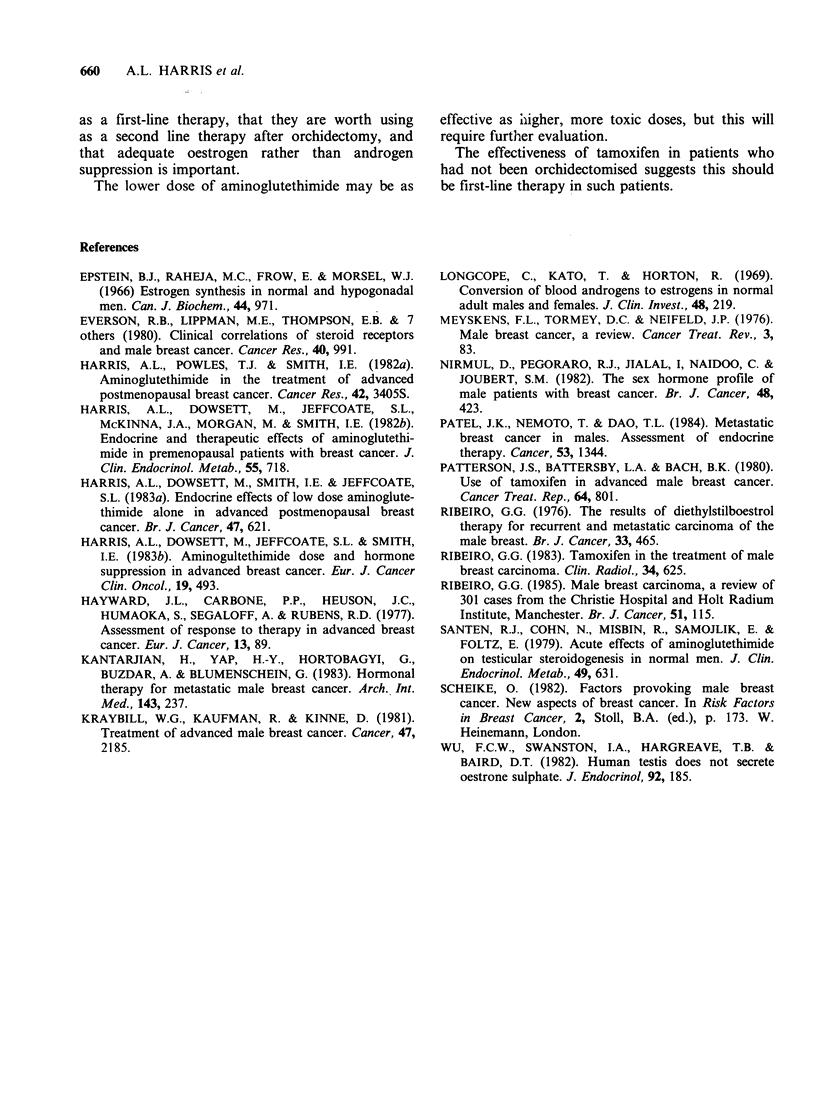# Role of aminoglutethimide in male breast cancer.

**DOI:** 10.1038/bjc.1986.223

**Published:** 1986-10

**Authors:** A. L. Harris, M. Dowsett, R. Stuart-Harris, I. E. Smith

## Abstract

**Images:**


*Br. J. Cancer* (1986), **54**, 657–660

### Role of aminoglutethimide in male breast cancer

A.L. Harris[1], M. Dowsett[2], R. Stuart-Harris[3] and I.E. Smith[3]

[1]*University Department of Clinical Oncology, Newcastle-upon-Tyne NE1 4LP;* [2]*Endocrine Department, Chelsea Hospital for Women, and* [3]*Medical Breast Unit, Royal Marsden Hospital, Fulham Road, London, UK.*

**Summary** Five men with advanced breast cancer were treated with aminoglutethimide (AG) plus replacement dose hydrocortisone. None of the 4 patients with intact testes responded, although 3 did so subsequently to tamoxifen. The previously orchidetomised patient responded to amminoglutethimide for 14 months. Oestrone and oestradiol were suppressed by AG in all patients, but not to the levels achieved by orchidectomy. AG produced substantial further oestrogen suppression in the orchidectomised patient and should only be used after orchidectomy.

Breast cancer in males is an uncommon disease. It accounts for 1% of all breast cancers and is frequently sensitive to hormone manipulation (Ribeiro, 1985). Breast cancer in men presents at a more advanced stage and in older age groups compared with women, but when matched for these prognostic variables, survival is similar (Scheike, 1982). Because of these clinical features, systemic endocrine therapy is commonly combined with local treatment, castration being a standard first line endocrine approach.

Recently, aminoglutethimide has been shown to be effective in postmenopausal women with breast cancer, the main mechanism of action being inhibition of the peripheral conversion of androgens to oestrogens by aromatase (Harris *et al.*, 1982*a*; 1983*a*). In men, three quarters of oestradiol produced results from peripheral aromatisation of testosterone (Epstein *et al.*, 1966; Longcope *et al.*, 1969; Wu *et al.*, 1982). Thus aminoglutethimide could potentially be useful either to avoid castration or as a second line endocrine therapy to suppress residual oestrogen production via androgens from the adrenal after castration.

### Patients and methods

Five men with advanced breast cancer were treated with aminoglutethimide and replacement dose hydrocortisone (20 mg twice daily). Four patients received the conventional dose of aminoglutethimide, 250 mg four times daily, and one received 125 mg twice daily.

Plasma samples were taken before treatment and at 2 weekly intervals for measurement of oestrone, oestradiol, testosterone and $\Delta^4$ androstenedione by previously described assays (Harris *et al.*, 1982*a,b*; 1983*a,b*).

Tumour response was assessed by standard UICC criteria (Hayward *et al.*, 1977).

Correspondence: A.L. Harris.
Received 3 March 1986; and in revised form, 4 June 1986

### Results

### *Clinical responses to aminoglutethimide and hydrocortisone*

The pretreatment characteristics are shown in Table I. Only 1 patient had been orchidectomised. He initially presented with secondary deposits in his axilla and a pleural effusion. Initial treatment was with orchidectomy plus chemotherapy with 5-fluorouracil (5FU), methotrexate (MTX) and vincristine (VCR) for eight courses. There was a partial response. He relapsed in the axilla again a year later and responded to ethinyloestradiol. After 3 years, a further local recurrence was excised. He relapsed 12 months later in the lung and bone and received aminoglutethimide. He responded to aminoglutethimide for 14 months. There was no response in the 4 patients with intact testes. This was not due to hormone resistance, since three of these patients responded very well to tamoxifen. Responses to tamoxifen are shown in Figures 1 and 2.

### *Endocrine effects of aminoglutethimide and hydrocortisone*

Oestrone and oestradiol levels were suppressed in all patients (Figure 3). However, in only 1 patient out of the 4 not orchidectomised, did oestrone fall below the basal oestrone level in the castrate patient. In none of the former 4 did oestradiol fall below the basal level in the castrate patient. In the

© The Macmillan Press Ltd., 1986

**Table I** Male breast cancer patients treated with aminoglutethimide

| *Patient no. and age* | *Previous treatment* | | | | | | | |
|---|---|---|---|---|---|---|---|---|
| | *Surgery* | *Radiotherapy* | *Endocrine* | *Chemotherapy* | *DFI** | *Sites of disease* | *Response to AG* | *Subsequent response to tamoxifen* |
| (1) 64 | Excision biopsy | — | Orchidectomy ethinyloestradiol | 5FU, MTX, VCR | 0 | Lung<br>Bone | PR<br>PR | — |
| (2) 36 | Mastectomy | — | — | | 4 yr | Lung<br>Bone | PD<br>PD | PR<br>PR |
| (3) 35 | Mastectomy axillary dissection | Local adjuvant | — | | 6 m | Lung | PD | PR |
| (4) 64 | Mastectomy | Local adjuvant | — | | 3 yr | Lung | PD | PR |
| (5) 79 | — | — | — | | 0 | Axillary local skin | PD | PD |

*Disease-free interval

**Figure 1** *Response of lung secondaries to tamoxifen.* Patient no. 5. Progressive lung secondaries on aminoglutethimide (a) tamoxifen response (b) film 7 months later than (a).

latter, basal oestrone and oestradiol levels were further suppressed.

Testosterone and androstenedione were not significantly affected.

### Discussion

A high frequency of oestrogen receptor positive tumours has been reported in male breast cancer, 85% in one series (Everson *et al.*, 1980). Farrow and Adair first reported a response to bilateral orchidectomy and this has been a standard first-line therapy. Recent series show a response rate of 32–

**Figure 2** *Response of rib secondary to tamoxifen.* Patient no. 2. Progressive 7th rib destruction on aminoglutethimide (a). Tamoxifen response (b) film 4 months later than (a).

**Figure 3** *Endocrine effects of aminoglutethimide in male patients with breast cancer.* Each symbol represents a different patient. Samples were taken before starting aminoglutethimide and at 2 weekly intervals. O——O, orchidectomised patient (no. 1).

48% (Patel *et al.*, 1984; Kraybill *et al.*, 1981). Castration removes both androgens and a major source of oestrogen precursors so it is not clear which is the major determinant of responses in male breast cancer. However, tamoxifen also produces responses in men who have not been castrated (25–66% response rate, Patterson *et al.*, 1980; Ribeiro, 1983), so antioestrogen effects are important. Stilboestrol has been used as a first-line therapy, but this will also suppress androgens (Ribeiro, 1976).

Adrenalectomy removes adrenal androgens which are the main residual source of both androgens and oestrogens after castration. Responses of 73–80% (14/19, 8/10) have been reported, all as second line after orchidectomy (Patel *et al.*, 1984; Meyskens *et al.*, 1976).

Corticosteroids alone produced a response of 43% after orchidectomy, presumably by suppressing residual adrenal androgen production and hence removing a source of oestrogens (Kantarjian *et al.*, 1983).

The endocrine data show that aminoglutethimide plus hydrocortisone suppresses peripheral oestrone and oestradiol levels.

The lower dose of aminoglutethimide plus hydrocortisone produced as much oestrone and oestradiol suppression as the conventional dose plus hydrocortisone. A similar effect of conventional doses has been shown in normal males (Santen *et al.*, 1979). However, this suppression was inadequate to produce a therapeutic response. Basal oestradiol levels in our series were in the same range as reported by Nirmul *et al.* (1982). The patients were not intrinsically resistant to endocrine therapy, since 3 out of 4 responded to tamoxifen. Aminoglutethimide did not suppress oestrogens to the level obtained by orchidectomy, thus suggesting a dose-response effect.

In the orchidectomised patient, a response was obtained and residual oestrogen levels were suppressed by more than 50%. There is one previous case of effective therapy with aminoglutethimide (Patel *et al.*, 1984) after orchidectomy.

Although these results are only on a small number of patients, they suggest that aminoglutethimide plus hydrocortisone should not be used as a first-line therapy, that they are worth using as a second line therapy after orchidectomy, and that adequate oestrogen rather than androgen suppression is important.

The lower dose of aminoglutethimide may be as effective as higher, more toxic doses, but this will require further evaluation.

The effectiveness of tamoxifen in patients who had not been orchidectomised suggests this should be first-line therapy in such patients.

### References

EPSTEIN, B.J., RAHEJA, M.C., FROW, E. & MORSEL, W.J. (1966) Estrogen synthesis in normal and hypogonadal men. *Can. J. Biochem.*, **44,** 971.

EVERSON, R.B., LIPPMAN, M.E., THOMPSON, E.B. & 7 others (1980). Clinical correlations of steroid receptors and male breast cancer. *Cancer Res.*, **40,** 991.

HARRIS, A.L., POWLES, T.J. & SMITH, I.E. (1982*a*). Aminoglutethimide in the treatment of advanced postmenopausal breast cancer. *Cancer Res.*, **42,** 3405S.

HARRIS, A.L., DOWSETT, M., JEFFCOATE, S.L., McKINNA, J.A., MORGAN, M. & SMITH, I.E. (1982*b*). Endocrine and therapeutic effects of aminoglutethimide in premenopausal patients with breast cancer. *J. Clin. Endocrinol. Metab.*, **55,** 718.

HARRIS, A.L., DOWSETT, M., SMITH, I.E. & JEFFCOATE, S.L. (1983*a*). Endocrine effects of low dose aminoglutethimide alone in advanced postmenopausal breast cancer. *Br. J. Cancer*, **47,** 621.

HARRIS, A.L., DOWSETT, M., JEFFCOATE, S.L. & SMITH, I.E. (1983*b*). Aminogultethimide dose and hormone suppression in advanced breast cancer. *Eur. J. Cancer Clin. Oncol.*, **19,** 493.

HAYWARD, J.L., CARBONE, P.P., HEUSON, J.C., HUMAOKA, S., SEGALOFF, A. & RUBENS, R.D. (1977). Assessment of response to therapy in advanced breast cancer. *Eur. J. Cancer*, **13,** 89.

KANTARJIAN, H., YAP, H.-Y., HORTOBAGYI, G., BUZDAR, A. & BLUMENSCHEIN, G. (1983). Hormonal therapy for metastatic male breast cancer. *Arch. Int. Med.*, **143,** 237.

KRAYBILL, W.G., KAUFMAN, R. & KINNE, D. (1981). Treatment of advanced male breast cancer. *Cancer*, **47,** 2185.

LONGCOPE, C., KATO, T. & HORTON, R. (1969). Conversion of blood androgens to estrogens in normal adult males and females. *J. Clin. Invest.*, **48,** 219.

MEYSKENS, F.L., TORMEY, D.C. & NEIFELD, J.P. (1976). Male breast cancer, a review. *Cancer Treat. Rev.*, **3,** 83.

NIRMUL, D., PEGORARO, R.J., JIALAL, I, NAIDOO, C. & JOUBERT, S.M. (1982). The sex hormone profile of male patients with breast cancer. *Br. J. Cancer*, **48,** 423.

PATEL, J.K., NEMOTO, T. & DAO, T.L. (1984). Metastatic breast cancer in males. Assessment of endocrine therapy. *Cancer*, **53,** 1344.

PATTERSON, J.S., BATTERSBY, L.A. & BACH, B.K. (1980). Use of tamoxifen in advanced male breast cancer. *Cancer Treat. Rep.*, **64,** 801.

RIBEIRO, G.G. (1976). The results of diethylstilboestrol therapy for recurrent and metastatic carcinoma of the male breast. *Br. J. Cancer*, **33,** 465.

RIBEIRO, G.G. (1983). Tamoxifen in the treatment of male breast carcinoma. *Clin. Radiol.*, **34,** 625.

RIBEIRO, G.G. (1985). Male breast carcinoma, a review of 301 cases from the Christie Hospital and Holt Radium Institute, Manchester. *Br. J. Cancer*, **51,** 115.

SANTEN, R.J., COHN, N., MISBIN, R., SAMOJLIK, E. & FOLTZ, E. (1979). Acute effects of aminoglutethimide on testicular steroidogenesis in normal men. *J. Clin. Endocrinol. Metab.*, **49,** 631.

SCHEIKE, O. (1982). Factors provoking male breast cancer. New aspects of breast cancer. In *Risk Factors in Breast Cancer*, **2,** Stoll, B.A. (ed.), p. 173. W. Heinemann, London.

WU, F.C.W., SWANSTON, I.A., HARGREAVE, T.B. & BAIRD, D.T. (1982). Human testis does not secrete oestrone sulphate. *J. Endocrinol*, **92,** 185.